# Molecular cloning and expression analysis of adiponectin and its receptors (AdipoR1 and AdipoR2) in the hypothalamus of the Huoyan goose during different stages of the egg-laying cycle

**DOI:** 10.1186/s12958-015-0085-1

**Published:** 2015-08-07

**Authors:** Zhongzan Cao, Juan Li, Lina Luo, Xiaoshuang Li, Mei Liu, Ming Gao, Yunhou Yin, Xinhong Luan

**Affiliations:** College of Animal Science and Veterinary Medicine, Shenyang Agricultural University, Shenyang, 110866 China; Guizhou Minzu University, Guiyang, 550025 China

**Keywords:** Huoyan goose, Adiponectin, Adiponectin receptor, Hypothalamus, Egg-laying cycle

## Abstract

**Background:**

Adiponectin and its receptors (AdipoR1 and AdipoR2) are novel endocrine systems that act at various levels to regulate metabolic homeostasis and reproductive processes. We cloned and characterized the cDNA of adiponectin and its receptors from the hypothalamus of the Huoyan goose to reveal the influence of these factors on the process of goose egg-laying. We also determined the mRNA and protein expression profiles during different stages of the egg-laying cycle.

**Methods:**

Hypothalamus tissues were obtained from 36 Huoyan geese in the pre-laying, early-laying, peak-laying, and ceased periods. The cDNA sequences of goose adiponectin and its receptors (AdipoR1 and AdipoR2) were cloned and characterized using the 5’-RACE and 3’-RACE methods. Multiple alignments and phylogenetic analyses of the deduced amino acid sequence were conducted using bioinformatics tools. The expression profiles of mRNA and protein in the hypothalamus during the pre-laying, early-laying, peak-laying and ceased periods were examined using real-time PCR (qRT-PCR) and Western blotting techniques.

**Results:**

The cDNA of adiponectin, AdipoR1 and AdipoR2 consisted of 738, 1131 and 1161 bp open reading frame encoding 245, 376 and 386 amino acids, respectively. The deduced amino acid sequence of goose adiponectin, as well as AdipoR1 and AdipoR2 showed a closer genetic relationship to the avian species than to other mammal species. The expression level of adiponectin mRNA and protein increased from the pre-laying period to the peak-laying period, reached its peak in the peak-laying period, and then decreased during the ceased period. Conversely, the expression levels of AdipoR1 and AdipoR2 mRNA and protein decreased in the early-laying period, peak-laying period, and ceased period compared with the pre-laying period.

**Conclusions:**

This study is the first to obtain full-length cDNA sequences of goose adiponectin and the genes of its receptors from the hypothalamus, and demonstrate that the egg-laying cycle affects the expression of the goose adiponectin system. Our results suggest the potential role of adiponectin as a key neuromodulator of reproductive functions.

**Electronic supplementary material:**

The online version of this article (doi:10.1186/s12958-015-0085-1) contains supplementary material, which is available to authorized users.

## Background

Adiponectin is one of adipocytokine hormones secreted primarily by adipocytes. It belongs to a family of proteins that contain sequences homologous to the C1q globular domain. Adiponectin consists of an N-terminal collagenous domain and C-terminal globular domain [1, 2]. The biological activity of this hormone is mediated via two distinct receptors, termed adiponectin receptor 1 (AdipoR1) and adiponectin receptor 2 (AdipoR2). Both receptors contain seven transmembrane domains with an extracellular carboxyl terminus and an intracellular amino terminus that are structurally and functionally distinct from G protein-coupled receptors. AdipoR1 shows high affinity for the globular form of adiponectin, and AdipoR2 has an intermediate binding affinity for both full-length and globular species [3, 4]. Activation of these cell membrane bound receptors can then activate the AMP activated protein kinase (AMPK), peroxisome proliferator activated receptor alpha (PPARα) and mitogen activated protein kinase (MAPK) [5]. Studies have suggested that the adiponectin, AdipoR1 and AdipoR2 genes are expressed in multiple tissues including the anterior pituitary gland, hypothalamus, testis and ovary in mammals and Avian [6, 7].

Adiponectin plays a dominant role in lipid and carbohydrate metabolism, stimulates fatty acid oxidation, decreases plasma triglycerides, and improves glucose metabolism by increasing insulin sensitivity [3]. Adiponectin also seems to be an important factor linking the regulation of reproductive processes [8]. Evidence has indicated that adiponectin influences the reproductive system by exerting central or peripheral effects on the hypothalamus-pituitary-gonadal (HPG) axis. The adiponectin receptors (AdipR1 and AdipR2) are expressed in mouse GT1-7 cells derived from hypothalamus neurons, and adiponectin inhibits GnRH secretion through the activation of AMP-activated protein kinase (AMPK) [9]. The expression of adiponectin and its receptors is also responsible for GnRH secretion in porcine hypothalamic structures including the medio-basal hypothalamus (MBH), preoptic area (POA) and median eminence (SME)[10]. Adiponectin could influence oxytocin-secreting neuron excitability and enhanced oxytocin secretion in the paraventricular nucleus of the hypothalamus (PVN) [11]. In addition, adiponectin seems to be involved in the autocrine/paracrine control of pituitary gonadotrophs and the secretion of LH, GH and FSH [12–14]. It was also described as a factor affecting ovarian steroidogenesis and oocyte maturation [15–17]. Therefore, the relation between expression in the hypothalamus, pituitary and gonadal adiponectin systems and the reproductive cycle has been investigated in porcine, cow, and chicken [15, 18, 19]. These results demonstrated that the adiponectin system may affect reproductive functions by controlling the hypothalamic-pituitary-gonadal axis and that the expression of adiponectin and the adiponectin receptors is dependent on the endocrine status of animals.

In poultry, the reproductive endocrine system and reproductive activity are strictly controlled by the hypothalamic-pituitary-gonadal axis [20]. The hypothalamus regulate reproduction by releasing neurohormones (gonadotropin-releasing hormones, GnRH) to the pituitary gland, and then the pituitary gland synthesizes and releases gonadotropins (luteinizing hormone, LH; follicle-stimulating hormone, FSH), which in turn act on the gonads to stimulate gametogenesis (spermatogenesis, oogenesis) and sex steroid hormones secretion (androgens, oestrogens, and progesterone). To our knowledge, there are no other reports regarding the expression changes of adiponectin, AdipoR1 and AdipoR2 in the hypothalamic-pituitary-gonadal axis of the goose during different egg-laying stages. Therefore, the current study was undertaken to clone the full-length cDNA of Huoyan goose adiponectin and its receptors by RACE (rapid amplification of cDNA ends), and identify its sequence characteristics. Subsequently, we aimed to determine the gene and protein expression profiles of these genes in the hypothalamus of the Huoyan goose during pre-laying, early-laying, peak-laying and ceased periods, with the use of real-time PCR and western blotting. These results provide a more complete understanding of the action of the adiponectin system with respect to the process of geese egg-laying.

## Methods

### Animal and tissue collection

Thirty-six Huoyan geese were selected randomly from two hundred geese on the Liaoning Huoyan goose stock breeding farm and raised according to the farm program. During the experiment, geese were fed ad libitum with rice grain and were supplemented with green grass or water plants whenever possible. Feed was given during the daytime when the geese were released into an open area outside the house. Huoyan geese become sexually mature at approximately 7 months of age and reach the peak egg-laying stage in the following year. In the current study, goslings were purchased in the fall of the year and become sexually mature during the summer of the following year. Nine geese were killed by exsanguination at the age of 6 months (pre-laying period), 9 months (early laying period), 12 months (peak-laying period), and 15 months (ceased period). The hypothalamus were quickly dissected, frozen in liquid nitrogen, and stored at −80 °C until total RNA extraction and protein were prepared. All experimental procedures were reviewed and approved by the animal welfare committee of the College of Animal Science and Veterinary Medicine of Shenyang Agricultural University (No. 2011036) and performed in accordance with the Regulations for the Administration of Affairs Concerning Experimental Animals (China, 1988) and EU Directive 2010/63/EU for animal experiments.

### Cloning and sequencing of goose adiponectin, AdipoR1 and AdipoR2

Total RNA was extracted using Trizol reagent (Invitrogen Corporation, Carlsbad, CA) following the manufacturer’s protocol. The quality of the RNA was determined by agarose gel electrophoresis and NanoDrop 8000 spectrophotometry (NanoDrop, Thermo Scientific). One microgram of RNA was reversely transcribed to cDNA using a PrimeScript®RT reagent Kit (TaKaRa, Dalian, China) in a total volume of 20 μl with 4.0 μl of 5 × PrimeScript®Buffer, 1.0 μl of PrimeScript®RT Enzyme Mix, 1.0 μl of Random 6 mers, 1.0 μl of oligo(dT)_18_ Primer and 9.0 μl of RNase Free H_2_O. Thermal cycling was performed for 15 min at 37 °C, and then 5 s at 85 °C. RT products were stored at 20 °C prior to RT-PCR. Specific PCR primer pairs used to amplify regions of the adiponectin, AdipoR1 and AdipoR2 cDNA sequences were designed by using Primer Premier 6.0 (Primer Biosoft International, Palo Alto, California, USA) according to the mRNA sequence of the Gallus gallus adiponectin AdipoR1 and Anas platyrhynchos AdipoR2 genes (NM_206991.1, NM_001031027.1 and XM_005010210.1). All of the primer pairs were synthesized commercially by Sangon Biotech Co., LTD (Shanghai, China). The primers are listed in Table 1. The 50 μl reaction consisted of 1 μl of cDNA, 8 μl of deoxynucleoside triphosphate Mix (2.5 mmol/L each dATP, dGTP, dCTP and dTTP), 2 μl of each primer (10 μmol/l), 5 μl of 10 × LA PCR Buffer, 0.5 μl of 5U/μL LA Taq™ (TaKaRa, Dalian, China), and 31.5 μl sterile MilliQ water. PCR conditions for amplification of adiponectin, AdipoR1, and AdipoR2 were 35 cycles consisting of denaturing at 94 °C for 30 s, specific annealing for 30 s, and extension at 72 °C for 90 s with an initial denaturing step at 94 °C for 5 min and a final extension step at 72 °C for 10 min. The annealing temperatures were 55, 60, and 50 °C for adiponectin, AdipoR1, and AdipoR2, respectively. The PCR products were gel-purified and ligated into the pMD-18-T vector (TaKaRa, Dalian, China), transformed into the competent E. coli DH5α competent cell. Positive clones containing the expected-size inserts were screened by colony PCR and then sequenced by Sangon Biotech Co., LTD, and its characteristics were determined using Basic Local Alignment Search Tool (nBLAST) at http://blast.ncbi.nlm.nih.gov/Blast.cgi.

**Table 1:** Primers used in this study

Primers purpose	Primer name	Primer sequence (5’-3’)
RT-PCR	Adiponectin-F	TCCTCCTTTGCTCACTGCT
	Adiponectin-R	CGGCCTTGTCCTTCTTGTA
	AdipoR1-F	AGGAGGAAGTTGTCCGTGTG
	AdipoR1-R	CTTCGAGTCCGTAGCGAAAC
	AdipoR2-F	TCAAGAGGAGGCAGGATAT
	AdipoR2-R	GGAACCAGATGTCACACTT
3’-RACE	Adiponectin-GSP3	AGAACCACTACGACGCCAGCACCG
	Adiponectin-NGSP3	ATGTACTACTTCGCCTACCACCTGACG
	AdipoR1-GSP3	CCACCATGCACTTCACCATCGCCGA
	AdipoR1-NGSP3	GCATCAGATCTTCCATGTGCTCGTG
	AdipoR2-GSP3	CCTGGGCATCGCAGCCATAATTGTC
	AdipoR2-NGSP3	AGATAGGCTGGCTGGCACTCATGGC
5’-RACE	Adiponectin-GSP5	CATCTTTCCCGTCCCTGC
	Adiponectin-NGSP5	CGGGCTGGGGATCTGGAG
	AdipoR1-GSP5	AGCGCCCTTCCCACACCTTA
	AdipoR1-NGSP5	CATCTTCTCCATGGCGTGGT
RACE	UPM-Long	CTAATACGACTCACTATAGGGCAAGCAGTGGTATCAACGCAGAGT
	UPM-Short	CTAATACGACTCACTATAGGGC
Real-time PCR	Adiponectin-S	AACGAGCAGAACCACTAC
	Adiponectin-A	CGCCTTGTCCTTCTTGTA
	AdipoR1-S	AAGTTGGATTATTCAGGAA
	AdipoR1-A	AATGGAGAGGTAGATGAG
	AdipoR2-S	ATACTGAACAAGGCCACTATTT
	AdipoR2-A	CACCTGAATGCCTTACTCTC
Internal control	18S rRNA-S	CGGACAGGATTGACAGATTGAG
	18S rRNA-A	GCCAGAGTCTCGTTCGTTAT

Based on the partial cDNA sequences of adiponectin, AdipoR1 and AdipoR2 obtained from the above RT-PCR reaction, specific primers were designed to amplify the full-length cDNA sequence of goose adiponectin, AdipoR1 and AdipoR2 (primers shown in Table 1) using the SMARTer™ RACE cDNA Amplification kit (Clontech Laboratories, CA, USA) according to the manufacturer’s instructions. The 3’- and 5’-end cDNA templates were synthesized using the 3’-CDS Primer A and 5’-CDS Primer A provided in the kit. Nested PCR was used in the 3’-RACE analysis. The first-round of PCR was performed in a total volume of 50 μl containing 2.5 μl of the first strand 3’- end cDNA template, 5.0 μl of 10× Advantage 2 PCR buffer, 1.0 μl of 10 mM dNTP Mix, 1.0 μl of 10 μM gene-specific primer GSP3, 5.0 μl of 10 × Universal Primer Mix (UPM; Clontech, USA), 34.5 μl of sterile deionized water, and 1.0 μl of 50 × Advantage 2 Polymerase Mix (Clontech, USA). Then, 1 μl of the PCR product was diluted to 1:50 and subsequently amplified with the NGSP3 and UPM as described above. For the 5’ RACE, a 5’- end cDNA template, SMARTer™ cDNA kit UPM and the gene-specific primer GSP5 were used for the first-round PCR. These amplified products were then subjected to a second round of nested PCR with the UPM and NGSP5. PCR amplification conditions for 3’ and 5’ RACE were as follows: 5 cycles at 94 °C for 30 s and 72 °C for 3 min; 5 cycles at 94 °C for 30 s, 70 °C for 30 s, and 72 °C for 3 min; 25 cycles at 94 °C for 30 s, 68 °C for 30 s, and 72 °C for 3 min; a final extension for 10 min at 72 °C; and then cooled to 4 °C.

The final PCR products were gel-purified and ligated into the pMD-18-T vector (TaKaRa, Dalian, China) and then transformed into the E. coli DH5α competent cell. Positive clones containing the expected-size inserts were screened using colony PCR and then sequenced by Sangon Biotech Co., LTD.

### Bioinformatic analysis

The data of DNA sequences were edited and analysed using Lasergene 7.0 software (DNA Star Inc., Madison, USA), and similarity analyses of nucleotide and protein sequences were carried out using the BLAST program from the NCBI (http://blast.ncbi.nlm.nih.gov/Blast.cgi). The open reading frame (ORF) was obtained using the ORF finder (http://www.ncbi.nlm.nih.gov/gorf/gorf.html), and the coding region sequences were translated into amino acid sequences using the sequence manipulation suite (SMS) tool (http://www.bio-soft.net/sms/index.html). The homologous conserved domains were identified using SMART (Simple Modular Architecture Research Tool, http://smart.embl-heidelberg.de). The molecular weight and isoelectric point of this predicted protein were analysed using the ExPASy ProtParam tool (http://web.expasy.org/protparam/). The PSORT II web-based program (http://psort.hgc.jp/form2.html) was used to predict the subcellular distribution of these proteins. The presence of transmembrane regions, phosphorylation sites, N-glycosylation sites and the secondary structure of these proteins were predicted using the TMHMM, version 2.0; NetPhos, version 2.0; NetNGlyc, version 1.0; and SOPMA web-based programs, respectively. Multiple alignments of the adiponectin, AdipoR1, and AdipoR2 sequences were performed with the ClustalX2 program [21] and the phylogenetic tree was constructed using the neighbour-joining (NJ) methods (bootstrap phylogeny test, 1000 replicates) with the MEGA 4.0 program [22].

### Quantitative Real-time RT-PCR

To evaluate changes in the gene expression of adiponectin, AdipoR1, and AdipoR2 in hypothalamus of Huoyan goose during different stages of the egg-laying cycle, quantitative real-time RT-PCR (qRT-PCR) was performed. The information for the primers used for qRT-PCR is listed in Table 1. Total RNA was extracted using TRIzol Reagent (Invitrogen Corporation, Carlsbad, CA) according to the manufacturer’s instructions. The concentration and purity of the RNA were measured as described above. Two micrograms of total RNA was reverse transcribed using PrimerScript® RT reagent Kit (TaKaRa, Dalian, China). Real-time PCR was carried out on the Bio-Rad iQ5 Real-time PCR Detection System and software (BIO-RAD, California, USA). Each 25 μl reaction volume contained 1 μl 10 μM (each) forward and reverse primers, 12.5 μl 2 × SYBR® Premix Ex Taq™ II (Takara, Dalian, China), and 2 μl cDNA products, and the final volume was adjusted using PCR-water. The following PCR program was used for amplification: 15 min at 95 °C, 40 cycles of denaturation at 95 °C for 10 s and annealing and extension at 60 °C for 30 s. The 18S rRNA was selected as an internal reference gene and the expression level of 18S rRNA was used to normalize the qRT-PCR results for each gene. Negative controls without a cDNA template were included in this experiment. Standard curve testing was performed using a series of 10-fold diluted samples, respectively, for each gene. The slopes of standard curves and PCR efficiency for these genes were calculated to confirm the preciseness and validity of the RT-qPCR data. Melting curves were analysed to ensure that a single PCR product was amplified for each pair of primers. Product purity was confirmed by electrophoresis. All samples were amplified in triplicate. Threshold and Ct (threshold cycle) values were determined automatically by the Bio-Rad iQ5 Real-time PCR Detection software using default parameters. The relative levels of expression for adiponectin, AdipoR1, and AdipoR2 were calculated relative to 18S rRNA using the 2^−ΔΔCt^ method [23].

### Western blotting analysis

Protein samples of hypothalamus tissues from the pre-laying, early-laying, peak-laying, and ceased groups were extracted and determined using kits according to the manufacturer’s instructions (Applygen Co., LTD. Beijing, China). Equivalent amounts of total protein were subjected to 12 % SDS-PAGE and then transferred to a nitrocellulose membrane. After blocking for 1 h at 37 °C, the membranes were incubated separately with rabbit Anti-Adiponectin antibody (bs-0471R, Beijing Biosynthesis Biotechnology Co., LTD), rabbit Anti-Adiponectin Receptor 1 antibody (bs-0610R, Beijing Biosynthesis Biotechnology Co., LTD), and rabbit anti-Adiponectin receptor 2 antibody (bs-0611R, Beijing Biosynthesis Biotechnology Co., LTD) overnight at 4 °C. The membranes were subsequently incubated with HRP-conjugated goat anti-rabbit antibody (bs-0295G-HRP, Beijing Biosynthesis Biotechnology Co., LTD) for 1 h at 37 °C. Finally, the bands were captured using a MicroChemi4.2 imaging system (DNR Bio-imaging Systems, Jerusalem, Israel), and densitometry analysis of protein bands was performed using GelQuant software (DNR Bio-imaging Systems, Jerusalem, Israel). β-actin (sc-47778, Santa Cruz Biotechnology, USA) was used as a reference protein to ensure equal loading. Triplicate experiments were performed for each sample.

### Statistical analysis

The mRNA level or protein abundance of adiponectin, AdipoR1, and AdipoR2 in the pre-laying period was assigned a value of 1. All data were analysed using SPSS 16.0 for Windows (SPSS Inc. Chicago, Illinois, USA). The data were analysed by one-way ANOVA, followed by a Tamhane’s T2 post hoc test. The results are expressed as the mean ± SEM. P < 0.05 was considered to be statistically significant.

## Results

### Cloning and characteristics of the adiponectin, AdipoR1 and AdipoR21 + cDNA

The full-length cDNA sequences of the goose adiponectin, AdipoR1 and AdipoR2 genes were obtained as described above and deposited in GenBank (GenBank Accession No. KP993199, KP993200, and KP993201), respectively. Adiponectin is 2221 bp in length and consists of a 55 bp 5’ UTR, a 738 bp ORF encoding 245 amino acids (Additional file 1: Figure S1), and a 1428 bp 3’ UTR. According to the prediction from ProtParam, the molecular mass of the goose adiponectin protein is 26.5416 kDa, and the theoretical isoelectric point is 5.19. Under the analysis of the deduced amino acid sequence by the SMART program, adiponectin contained one C1Q domain (Complement component C1q domain) from amino acid residues 105 to 241. The subcellular distribution of the adiponectin protein was predicted to be 66.7 % extracellular, 22.2 % vacuolar, and 11.1 % endoplasmic reticulum. Eleven putative phosphorylation sites were identified in the adiponectin protein, which included five serine residues (Ser75, Ser115, Ser173, Ser205, and Ser231), three threonine residue (Thr33, Thr84, and Thr119), and three tyrosine residues (Tyr108, Tyr142, and Tyr224). The secondary structure of the adiponectin protein was predicted to consist of 2.45 % α-helix, 27.76 % extended strand, 9.39 % β-turn, and 60.41 % random coil. No N-glycosylation sites were found in the transmembrane domain.

AdipoR1 contains 1465 bp with a 206 bp 5’ UTR, an 1131 bp ORF encoding 376 amino acids (Additional file 2: Figure S2), and a 128 bp 3′ UTR. The predicted molecular mass of the goose AdipoR1 protein is 42.2623 kDa, and the theoretical isoelectric point is 6.92. The subcellular distribution of the AdipoR1 protein was predicted to be 55.6 % in endoplasmic reticulum, 33.3 % in plasma membrane, and 11.1 % in mitochondria. As in mammals, seven transmembrane domains were found from the amino acids residues 136 to 158, 173 to 195, 208 to 227, 237 to 256, 269 to 288, 298 to 320, and 333 to 355. Ten putative phosphorylation sites were identified in the AdipoR1 protein, which included five serine residues (Ser48, Ser198, Ser202, Ser206, and Ser211), two threonine residues (Thr261 and Thr267), and three tyrosine residues (Tyr86, Tyr110, and Tyr210). The secondary structure of the AdipoR1 protein was predicted to consist of 38.3 % α-helix, 21.28 % extended strand, 9.04 % β-turn, and 31.38 % random coil. No N-glycosylation site was found.

AdipoR2 is 3820 bp in length and consists of a 41 bp 5’ UTR, a 1161 bp ORF encoding 386 amino acids (Additional file 3: Figure S3), and a 2618 bp 3’ UTR. According to the prediction of the ProtParam, the molecular mass of the goose AdipoR2 protein is 43.5533 kDa, and the theoretical isoelectric point is 5.6. The subcellular distribution of the AdipoR2 protein was predicted to be 65.2 % in the plasma membrane, 26.1 % in the endoplasmic reticulum, 4.3 % in the Golgi, and 4.3 % in the mitochondria. As expected, seven transmembrane domains were also found from the amino acid residues 145 to 167, 180 to 202, 217 to 239, 246 to 265, 275 to 297, 310 to 332, and 342 to 364. Thirteen putative phosphorylation sites were identified in the AdipoR2 protein, which included ten serine residues (Ser12, Ser34, Ser38, Ser39, Ser52, Ser57, Ser58, Ser67, Ser215, and Ser220), one threonine residue (Thr374), and two tyrosine residues (Tyr119 and Tyr219). One putative N-glycosylation site located at amino acid positions 170 was identified. The secondary structure of the AdipoR2 protein was predicted to consist of 35.23 % α-helix, 23.06 % extended strand, 8.29 % β-turn, and 33.42 % random coil.

### Sequence alignment and phylogenetic analysis

The amino acid sequence similarities between Huoyan goose adiponectin, AdipoR1 and AdipoR2 and those of other representative species were investigated by multiple sequence alignment using the ClustalX2 program (See Additional file 4: Figure S4, Additional file 5: Figure S5 and Additional file 6: Figure S6). The overall percent identity among these sequences is shown in Table 2; the deduced amino acid sequence of Huoyan goose adiponectin shared 96 %, 99 %, and 85 % homology with duck (Anas platyrhynchos), chicken (Gallus gallus), and turkey (Meleagris gallopavo) adiponectin, respectively, and 70-73 % similarity with Sheep (Ovis aries), Dog (Canis lupus familiaris), Cat (Felis catus), human (Homo sapiens), turkey (Meleagris gallopavo), mouse (Mus musculus), pig (Sus scrofa). Unlike adiponectin, the deduced amino acid sequence of goose AdipoR1 and AdipoR2 are highly similar to that of mammalian AdipoR1 (91-99 %) and AdipoR2 (82-99 %).

**Table 2:** Amino acid sequence identities of adiponectin, AdipoR1 and AdipoR2 between the Huoyan goose and other vertebrate species

Gene	Matched species	GenBank accession no.	% Identity
adiponectin	Duck (*Anas platyrhynchos*)	ADA68839.1	96
	Sheep (*Ovis aries*)	AHV91023.1	70
	Dog (*Canis lupus familiaris*)	BAD15362.1	71
	Cat (*Felis catus*)	BAF52934.1	73
	Chicken (*Gallus gallus*)	AAX40986.1	99
	Human (*Homo sapiens*)	NP_004788.1	73
	Turkey (*Meleagris gallopavo*)	XP_010714799.1	85
	Mouse (*Mus musculus*)	NP_033735.3	71
	Pig (*Sus scrofa*)	ABQ95350.1	71
AdipoR1	Duck (*Anas platyrhynchos*)	ABI49513.2	99
	Sheep (*Ovis aries*)	AHV91022.1	91
	Dog (*Canis lupus familiaris*)	XP_005622230.1	90
	Cat (*Felis catus*)	NP_001128153.1	90
	Chicken (*Gallus gallus*)	NP_001026198.1	97
	Human (*Homo sapiens*)	NP_001277558.1	91
	Zebra finch (*Taeniopygia guttata*)	XP_002198547.1	95
	Mouse (*Mus musculus*)	NP_082596.2	91
	Pig (*Sus scrofa*)	NP_001007194.1	91
AdipoR2	Duck (*Anas platyrhynchos*)	ABC75392.1	99
	Bovine (*Bos taurus*)	NP_001035589.1	84
	Dog (*Canis lupus familiaris*)	XP_005637426	84
	Cat (*Felis catus*)	XP_011282054.1	83
	Chicken (*Gallus gallus*)	NP_001007855.1	97
	Human (*Homo sapiens*)	NP_078827.2	84
	Turkey (*Meleagris gallopavo*)	XP_003202489.1	97
	Mouse (*Mus musculus*)	NP_932102.2	84
	Pig (*Sus scrofa*)	NP_001007193.1	82

A phylogenetic tree was constructed using the MEGA program based on the amino acid sequences of Huoyan goose adiponectin, AdipoR1 and AdipoR2 and the other species previously mentioned (See Additional file 7: Figure S7, Additional file 8: Figure S8 and Additional file 9: Figure S9). They were clustered into two subgroups consisting of avian species (including goose, duck, turkey, zebra finch, and chicken) in one group and mammalian species in the other. The phylogenetic tree indicated that the deduced goose adiponectin, and AdipoR1 and AdipoR2 proteins showed a closer genetic relationship to the avian species than to the mammal species.

### Expression profile of adiponectin, AdipoR1 and AdipoR2 mRNA in hypothalamus of Huoyan goose during different stages of the egg-laying cycle

**Fig. 1:**
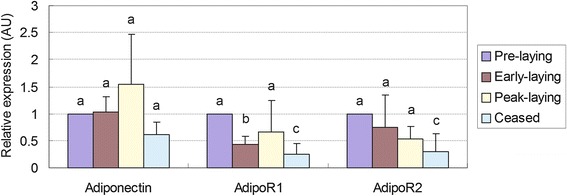
Relative expression of adiponectin, AdipoR1 and AdipoR2 mRNA in the hypothalamus of Huoyan geese during different stages of the egg-laying cycle. The expression levels of adiponectin, AdipoR1 and AdipoR2 were normalized to 18S rRNA. The expression levels, calculated by the 2^−ΔΔCt^ method, are presented as arbitrary units (AU). The presented values are the means ± SEM. The data were analysed by ANOVA followed by Tamhane’s T2 test post hoc test. Bars with different superscripts are significantly different (*P* < 0.05).

The mRNA levels of adiponectin, AdipoR1 and AdipoR2 in the hypothalamus of Huoyan geese during pre-laying, early-laying, peak-laying, and ceased periods were determined with qRT-PCR. As shown in Fig. 1, the expression of adiponectin mRNA increased from the pre-laying period to the peak-laying period, reached its peak during the peak-laying period, and then decreased and to the lowest level of expression during the ceased period. Conversely, the expression levels of AdipoR1 and AdipoR2 mRNA in the early-laying, peak-laying, and ceased periods were lower compared with the pre-laying period.

### Expression profile of adiponectin, AdipoR1 and AdipoR2 proteins in hypothalamus of Huoyan goose during different stages of the egg-laying cycle

**Fig. 2:**
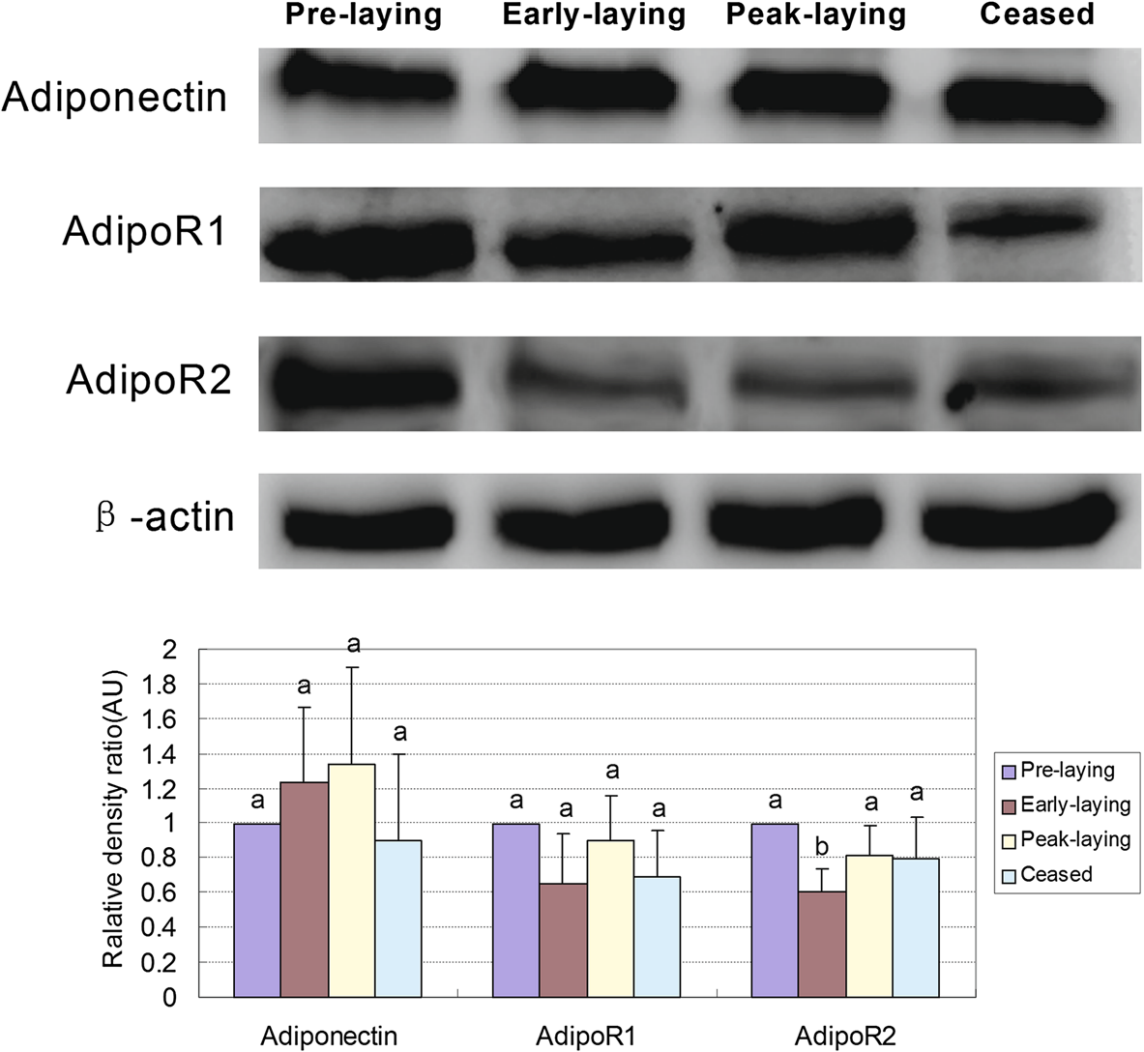
Relative expressions of adiponectin, AdipoR1 and AdipoR2 protein in the hypothalamus of Huoyan geese during different stages of the egg-laying cycle. A comparison of adiponectin, AdipoR1 and AdipoR2 protein content were determined by western blotting analysis. Protein band density was analysed with GelQuant software. Beta-Actin was used as the internal control. Upper panels: representative immunoblots. Lower panels: densitometric analysis of adiponectin, AdipoR1 and AdipoR2 proteins relative to actin protein. Values are expressed as the mean ± SEM of arbitrary optical density units. Data were analysed by ANOVA followed by Tamhane’s T2 test post hoc test. Bars with different superscripts are significantly different (*P* < 0.05).

Western blotting was used to analyse the protein expression of adiponectin, AdipoR1 and AdipoR2 in the hypothalamus of Huoyan geese during different egg-laying stages. Similar to the qRT-PCR results, the expression level of the adiponectin protein was the highest during the peak-laying period from pre-laying period, and decreased in ceased period. The AdipoR1 and AdipoR2 protein concentrations were highest during pre-laying period (Fig. 2).

## Discussion

Our study is the first to clone and characterize the cDNA and amino acid sequences of goose adiponectin and its receptors (AdipoR1 and AdipoR2) and compare the expression levels of these gene transcripts and proteins in the hypothalamus of Huoyan geese during different stages of the egg-laying cycle. We observed that adiponectin and its receptors were expressed in the hypothalamus tissues of Huoyan geese at both the mRNA and protein levels. Additionally the stage of the egg-laying cycle affected the abundance of mRNAs and proteins of both adiponectin and its receptors, which varied throughout the egg-laying cycle. We hypothesize these changes in expression might be related to the egg-laying process in birds.

First, hormones and other physiological conditions including energy metabolism strictly control the egg-production process in birds, from follicle maturation to ovulation. Moreover, it has long been recognized that female reproductive success depends largely on nutritional status and energy balance [24]. AMP-activated protein kinase (AMPK) is a master regulator of cellular and systematic energy homeostasis. Decreased AMPK activity in the hypothalamus leads to reduced food intake and increased energy expenditure [25]. Adiponectin was shown to stimulate fatty acid oxidation and enhance glucose uptake through the activation of AMP-activated protein kinase (AMPK) in the peripheral tissues, and activate AMPK via its receptor in the hypothalamus, stimulating food intake and decreasing energy expenditure [26, 27]. Recent studies suggest that increased expression of adiponectin depends on high nutritional status based on increased food intake [28]. Accordingly low circulating adiponectin levels were observed during the fasting period [29]. For avian species, adiponectin is likely to play a dominant role in carbohydrate and lipid metabolism because of the requirement to maintain a very high blood glucose concentration and lipid synthesis [30]. In our study, the varied expression level of adiponectin and its receptors throughout the egg-laying cycle may correlate with food availability and metabolic status during different egg-laying stages.

Second, laying performance is determined by the number of follicles destined for ovulation and the capacity of the oviduct to transform the ova into a hard-shelled egg. The development of follicles and ovulation are dependent on gonadotropins including luteinizing hormone (LH) and follicle-stimulating hormone (FSH) secreted by the pituitary, which in turn is regulated by the hypothalamic gonadotropin releasing hormones (GnRH) (Kuo et al., 2005). An increasing amount of evidence suggests that multiple hormones associated with reproductive processes could be responsible for the complex regulation of the adiponectin system. The presence of adiponectin and its receptors in the human, pig, rat, murine, and chicken hypothalamus and pituitary suggests that adiponectin may be a factor in modulating the secretory functions of the central branches of hormonal axes and, indirectly, also their peripheral branches, including the HPG axis [12–14, 18, 31, 32]. Adiponectin can exert autocrine/paracrine effects on GnRH synthesis and the pituitary secretory functions and may then indirectly affect gonadal functions. Adiponectin was found to regulate GnRH secretions by activating the AMPK pathway and inhibiting the ERK pathway in vivo [33] and was also observed to influence growth hormone and LH secretion in vitro by the isolated anterior pituitary cells of rats [13]. In addition, in isolated anterior (AP) and posterior (NP) pituitary cells of pigs during different stages of the estrous cycle, adiponectin affected GnRH- and/or insulin-induced LH and FSH output in a manner dependent on the phase of the estrous cycle [34]. Furthermore, adiponectin also locally affects ovarian steroidogenesis and stimulated secretion of steroid hormones by regulating transport of total cholesterol, precursor for steroidogenesis in ovary. In adiponectin treated porcine granulosa cells from medium follicles of prepubertal gilts, higher levels of StAR and a lower expression of the P450 aromatase gene were observed [16]. Likewise, regulation of the expression of these steroidogenic genes (Cyp11a1, StAR, and Cyp19a1) by adiponectin also has been observed in the rat, bovine and human ovary [17, 35]. These finding suggest that adiponectin might affect steroidogenesis in ovarian cells through the regulation of steroidogenic gene expressions as well. Notably, Anuradha et al. revealed that a low dose of adiponectin treatment caused a significant increase in circulating progesterone and oestradiol concentrations in C. sphinx, whereas a high dose of adiponectin caused a significant increase in oestradiol levels, but only a marginal increase in the progesterone level [36]. Edmond P. et al. demonstrated that adiponectin was able to augment the human Chorionic Gonadotrophin (hCG)-stimulated activity of the 3βHSD enzyme and resulted in an acute increase in progesterone production, this suggested adiponectin may play a role in the rise in progesterone production approaching and following ovulation [5]. In chicken granulosa cells, adiponectin alone increased insulin-like growth factor 1 (IGF-1)-induced progesterone secretion, but in combination with LH or follicle-stimulating hormone (FSH), adiponectin reduced progesterone secretion in cultured granulosa cells [15]. We hypothesize that the egg-laying process of bird is regulated by a variety of reproductive hormones, and our findings suggest that the adiponectin system may affect egg-laying performance through changes in endocrine functions. Additionally the adiponectin system could also affect gonadal functions by controlling the secretory activity of the hypothalamus and pituitary. In our previous report, the mRNA expression of another gene, synaptotagmin-1 (syt1), was also found to change in a manner similar to adiponectin in the same animal experiment. As a major transducer of Ca^2+^ signalling for dense-core vesicle exocytosis in neuroendocrine cells, syt1 can regulate the secretion of neurotransmitters and hormones of the hypothalamus and pituitary, including those relevant to reproduction [37]. These consistent findings require further investigation to determine the mechanism of action.

Finally, the synthesis and expression of adiponectin and its receptors are probably hormonally controlled and correlated with the animals’ hormonal milieu. Except for the expression inhibition of adiponectin mRNA by GnRH in immortalized LbT2 pituitary gonadotroph cells and primary pituitary cells of male rats, the progesterone level was found to be positively correlated with the adiponectin expression pattern [38]. Similar findings were reported by Caminos et al. who noted that progesterone had a stimulating effect on AdipoR2 gene expression [39]. Tan et al. observed that other gonadal steroids such as oestradiol and testosterone increase the mRNA and protein levels of the AdipoRs in cultured human adipocytes [40]. The reproductive cycle is controlled by FSH or LH, in bovine theca interna cells, and LH increased the concentrations of AdipoR2 mRNAs, and may be involved in the regulation of adiponectin receptor expression[17]. In addition, adiponectin may conduct its action through up- and/or downregulation of its own receptors, and could be an important factor in modulating its own receptor levels. Rodriguez-Pacheco et al. observed a significant decrease in AdipoR1 mRNA levels and an increase in AdipoR2 mRNA concentrations was noted in cultures of rat pituitary cells exposed to 10^–8^ M and 10^–7^ M of adipokine for 24 h, respectively [13]. Contrary results were presented by Caminos et al. who observed a suppressive effect of adiponectin on AdipoR2 mRNA expression in cultured human placenta explants [39]. Similar to the aforementioned findings, our study revealed the differential expression of adiponectin and its receptors from the pre-laying period to the ceased period. One possible explanation for this difference in accordance with egg-laying performance may be due to changes in sex hormone levels throughout the egg-laying cycle.

Collectively, the varied expression profiles of adiponectin, AdipoR1 and AdipoR2 suggests that adiponectin improves glucose utilization and regulates energy balance in response to nutritional states of the whole-body during different stages of the egg-laying cycle and subsequently determines the release of GnRH or gonadotropin to influence reproduction. The idea that the adiponectin system may function in the ovary during steroidogenesis and maturation of the oocyte is also supported.

## Conclusions

Our study was the first experiment to demonstrate the presence of both the mRNA and protein of adiponectin and its receptors in the Huoyan goose hypothalamus, and the effect of different stages of the egg-laying cycle on their expression. The variations in the expression levels of adiponectin and its receptor in the hypothalamus could be attributed to the influence of multiple hormones controlling reproductive processes. The results of this study suggested a role for adiponectin as a key neuromodulator of reproductive functions, which might operate as an endocrine integrator linking metabolism and gonadal function.

## Additional files

Additional file 1: Figure S1. Nucleotide and deduced amino acid sequences of adiponectin. The nucleotide (black) and deduced amino acid (blue) sequences are shown and numbered on the left. The nucleotide sequence is numbered from the 5’ end. The first methionine (M) is the first deduced amino acid. The C1Q domain (amino acids 105–241) is shaded. The start codons (ATG) and the stop codons (TAA) are marked in bold red. (TIFF 315 kb) Additional file 2: Figure S2. Nucleotide and deduced amino acid sequences of AdipoR1. The nucleotide (black) and deduced amino acid (blue) sequences are shown and numbered on the left. The nucleotide sequence is numbered from the 5’ end. The first methionine (M) is the first deduced amino acid. The start codons (ATG) and the stop codons (TAA) are marked in bold red. (TIFF 727 kb) Additional file 3: Figure S3. Nucleotide and deduced amino acid sequences of AdipoR2. The nucleotide (black) and deduced amino acid (blue) sequences are shown and numbered on the left. The nucleotide sequence is numbered from the 5’ end. The first methionine (M) is the first deduced amino acid. The start codons (ATG) and the stop codons (TAA) are marked in bold red. (TIFF 741 kb) Additional file 4: Figure S4. Multiple amino acid sequence alignment of the Huoyan goose adiponectin protein with other vertebrate species. The colour black denotes 100 % conserved sequences, and the colour grey indicates non-conservative sequences. Gaps (−) were introduced to maximize the alignment. Sequences for the alignment were obtained from GenBank (accession numbers are in brackets): Anas platyrhynchos (ADA68839.1); Ovis aries (AHV91023.1); Canis lupus familiaris (BAD15362.1); Felis catus (BAF52934.1); Gallus (AAX40986.1); Homo sapiens (NP_004788.1); Meleagris gallopavo (XP_010714799.1); Mus musculus (NP_033735.3); and Sus scrofa (ABQ95350.1). (TIFF 3483 kb) Additional file 5: Figure S5. Multiple amino acid sequence alignment of the Huoyan goose AdipoR1 protein with other vertebrate species. The colour black denotes 100 % conserved sequences, and the colour grey indicates non-conservative sequences. Gaps (−) were introduced to maximize the alignment. Sequences for the alignment were obtained from GenBank (accession numbers are in brackets): Anas platyrhynchos (ABI49513.2); Ovis aries (AHV91022.1); Canis lupus familiaris (XP_005622230.1); Felis catus (NP_001128153.1); Gallus (NP_001026198.1); Homo sapiens (NP_001277558.1); Taeniopygia guttata (XP_002198547.1); Mus musculus (NP_082596.2); and Sus scrofa (NP_001007194.1). (TIFF 4230 kb) Additional file 6: Figure S6. Multiple amino acid sequence alignment of the Huoyan goose AdipoR2 protein with other vertebrate species. The colour black denotes 100 % conserved sequences, and the colour grey indicates non-conservative sequences. Gaps (−) were introduced to maximize the alignment. Sequences for the alignment were obtained from GenBank (accession numbers are in brackets): Anas platyrhynchos (ABC75392.1); Bos taurus (NP_001035589.1); Canis lupus familiaris (XP_005637426); Felis catus (XP_011282054.1); Gallus (NP_001007855.1); Homo sapiens (NP_078827.2); Meleagris gallopavo (XP_003202489.1); Mus musculus (NP_932102.2); and Sus scrofa (NP_001007193.1). (TIFF 5171 kb) Additional file 7: Figure S7. The phylogenetic tree of adiponectin protein was constructed using the neighbour-joining method with MEGA4. Amino acid sequences of adiponectin for these species were downloaded from the protein database of the NCBI. Their corresponding accession numbers are the same as those given in Table 2. The number at the branches denotes the bootstrap majority consensus values on 1000 replicates; the branch lengths represent the relative genetic distances among these species. (TIFF 7463 kb) Additional file 8: Figure S8. The phylogenetic tree of AdipoR1 protein was constructed using the neighbour-joining method with MEGA4. The amino acid sequences of adiponectin for these species were downloaded from the NCBI protein database. Their corresponding accession numbers are shown in Table 2. The number at the branches denotes the bootstrap majority consensus values on 1000 replicates; the branch lengths represent the relative genetic distances among these species. (TIFF 7504 kb) Additional file 9: Figure S9. The phylogenetic tree of the AdipoR2 protein was constructed using the neighbour-joining method with MEGA4. The amino acid sequences of adiponectin for these species were downloaded from the NCBI protein database. Their corresponding accession numbers are the same as those given in Table 2. The number at the branches denotes the bootstrap majority consensus values on 1000 replicates; the branch lengths represent the relative genetic distances among these species. (TIFF 7477 kb)

## Abbreviations

aa: Amino acid(s)
bp: Base pair(s)
cDNA: DNA complementary to RNA
kDa: Kilodalton
mRNA: Messenger RNA
ORF: Open reading frame
PCR: Polymerase chain reaction
RACE: Rapid-amplification of cDNA ends
rRNA: Ribosomal RNA
UTR: Untranslated Regions
